# Trends in Alcohol Consumption for Korean Adults from 1998 to 2018: Korea National Health and Nutritional Examination Survey

**DOI:** 10.3390/nu13020609

**Published:** 2021-02-13

**Authors:** Sang Young Kim, Hyun Ja Kim

**Affiliations:** Department of Food and Nutrition, Gangneung-Wonju National University, 7 Jukheon-gil, Gangneung-si, Gangwon-do 25457, Korea; sangaustin@gmail.com

**Keywords:** alcohol, alcoholic beverage, trend, KNHANES

## Abstract

Drinking culture has been well developed in Korea. This research assessed trends in daily pure alcohol consumption over time and examined its trends regarding socio-demographic variables and alcoholic beverage types. We used data from the 1998–2018 Korea National Health and Nutrition Examination Survey. A total of 87,623 adults aged ≥ 19 years were included. Alcohol intake was assessed via 24-h dietary recall, and pure alcohol content was calculated according to alcoholic beverage type. Daily alcohol consumption increased from 8.37 g in 1998 to 14.98 g in 2016–2018 (*p* for trend < 0.001). The degree of the increasing trend was higher for women (2.09 g to 5.79 g) than men (14.78 g to 23.94 g) from 1998 to 2016–2018. Alcohol intake was highest in men aged 30–49 years and women aged 19–29 years. Moreover, the change of the rising trend in alcohol consumption according to high socioeconomic factors was more pronounced than the other variables. Lastly, the alcohol intake from soju and beer was dominant in alcohol consumption and escalated over time. The total daily alcohol intake increased about two times during 21 years in Korea, and the trends varied according to socio-demographic status.

## 1. Introduction

Alcohol consumption is prevalent and contributes to a critical part of entertainment culture in Korea. Although many studies have reported that moderate alcohol consumption can be beneficial to health, heavy drinking is well known to lead to adverse health consequences [1]. In recent years, recommendations regarding alcohol consumption have been updated and now no longer suggest even mild drinking. A study on alcohol consumption in 195 countries worldwide recommended that the safest level of alcohol intake is non-drinking, and consuming zero drinks per day minimizes overall health risks [2]. In addition, the national cancer prevention guidelines, published in 2016 by the National Cancer Center in Korea, suggested that individuals avoid even light alcohol consumption of one or two drinks per day to prevent cancer [3]. This recommendation was originally different, limiting alcohol to two alcoholic drinks (approximately 20 g of pure alcohol [4]) per day; however, it was revised based on the results that constant light drinking can increase cancer risks. In addition, numerous sources and guidance have been disseminated about alcohol intake, including mass media campaigns globally, to urge individuals to reduce alcohol intake and deliver alcohol-related health information [5]. Despite efforts to decrease alcohol intake, an increasing trend has been observed worldwide for alcohol consumption per capita over time from 5.5 L in 2005 to 6.4 L in 2016. This estimate was based on the amount of alcohol using sale and tax information according to a report by the World Health Organization [6]. In Korea, people consumed a remarkable amount of alcohol, with an average of 10.2 L per year in 2016, the highest level among Asian countries after Laos with 10.4 L [6]. Moreover, according to the Korea National Health and Nutrition Examination Survey (KNHANES), the rate of drinking at least once per month for Korean adults tended to increase from 54.6% in 2005 to 60.6% in 2018 [7].

Thus, it is important to examine trends in daily average alcohol consumption, and its trend changes by socio-demographic factors of the Korean population as alcohol guidelines can be established regarding the characteristics of people. Moreover, it is critical to identify trends in alcohol consumption according to types of alcoholic beverages over time, since the risk of diseases can be different based on alcoholic drink types.

This research investigated the actual daily intake of pure alcohol as grams for the first time, and observed the trend changes from 1998 to 2018 for the representative Korean adult population. Furthermore, this study examined trends in alcohol consumption regarding socio-demographic variables and various types of alcohol in Korea.

## 2. Materials and Methods

### 2.1. Study Design and Participants

This research utilized data sets from the 1998–2018 KNHANES [8]. The Korea Centers for Disease Control and Prevention (KCDC) of the Ministry of Health and Welfare in Korea initiated this repeated cross-sectional survey to observe the health and nutrition status of the Korean population. This survey consists of three major components: health examination, health interview, and nutrition survey. There are currently 15 data sets available in the 1998, 2001, 2005, and 2007–2018 KNHANES. The first three data sets were collected triennially during two or three months; however, this information did not reflect seasonal variations. Thus, data started being collected on an annual basis from 2007, and data collection continued throughout the year. This research used all the available data sets and was separated into seven categories, grouping three years as a set starting from 2007: KNHANES I (1998), KNHANES II (2001), KNHANES III (2005), KNHANES IV (2007–2009), KNHANES V (2010–2012), KNHANES VI (2013–2015), and KNHANES VII (2016–2018). Written informed consent was obtained from each participant and the KCDC Institutional Review Board (IRB) approved the survey procedure (2007-02CON-04-P, 2008-04EXP-01-C, 2009-01CON-03-2C, 2010-02CON-21-C, 2011-02CON-06-C, 2012-01EXP-01-2C, 2013-07CON-03-4C, 2013-12EXP-03-5C, waived from 2015 to 2017, and 2018-01-03-P-A). This research extracted the adult population aged ≥ 19 years who were able to drink legally in Korea, and included a total of 87,623 participants (37,249 men and 50,374 women).

### 2.2. General Characteristics

Korean adults were classified into four age categories: 19–29, 30–49, 50–64, and ≥65 years. Education levels were grouped as those who attended middle school or lower, high school, and college or higher, and income was divided into low, middle-low, middle-high, and high. Occupations were categorized as manual (professional, clerical, sales, and service positions), non-manual (agriculture, forestry, fishery, technician, and labor workers), and unemployed (including housewives and students). Regions were defined as urban (cities as *dong*) and rural (towns as *eup* or townships as *myeon*) areas.

### 2.3. Alcohol Intake Assessment

Trained dietitians administered the survey via face-to-face interviews in which participants were asked about food and alcohol intake for the past 24 h. The quantity and type of alcoholic beverages were surveyed via a single 24-h dietary recall, consisting of open-ended questionnaires. The amount of pure alcohol was calculated based on different types of alcoholic beverages as classified into seven groups: beer, makgeolli (a traditional Korean rice wine), wine, cheongju (a traditional Korean clear refined rice wine), soju (a Korean distilled spirit), liquor, and other (including cocktail and champagne). Alcohol content was specified as a percentage; for example, the amount of pure alcohol in beer was calculated as 4.5%, 6.0% for makgeolli, 12.0% for wine, 16.0% for cheongju, and 40.0% for liquor. However, the amount of pure alcohol in soju was considered separately by year since the percentage decreased over time, based on information of the soju industry; specifically, 25.0% in 1998, 2001, and 2005, 19.5% in 2007–2011, and 19.0% in 2012–2018.

### 2.4. Statistical Analysis 

To be representative of the Korean population, all the analyses applied strata, cluster, and nutrition survey weights for each sample [9]. These sampling weights were created considering information such as the non-response rate of survey participants and post-stratification. Continuous and categorical variables were described as mean values ± standard errors and percentages (standard errors), respectively. Mean values were age-standardized (except information on age and age group), and created according to the 2005 Korea census as a reference. Trend analyses were performed with adjustment for age via multivariate linear regressions for continuous variables and multivariate logistic regressions for categorical variables. In more detail, a multivariate linear regression model included each KNHANES set from I to VII as the independent variable and means of alcohol consumption as the dependent variable to calculate a regression coefficient. Statistical significance was tested using two-sided values < 0.05. SAS software version 9.4 (SAS Institute, Cary, NC, USA) with PROC SURVEY was used since the survey was designed as complex sample data.

## 3. Results

### 3.1. General Characteristics

Trends in general characteristics are illustrated in Table 1. A total number of participants was 7501 in 1998 including 3480 men (46.39%) and 4021 women (53.61%), and 16,854 in 2016–2018 including 7165 men (42.51%) and 9689 women (57.49%). There was a significant increase in mean age from 41.28 ± 0.33 years in 1998 to 47.28 ± 0.24 years in 2016–2018 (*p* for trend < 0.001). There was a significant rise of approximately two times in the percentage of the population with an education level of college or higher (*p* for trend < 0.001). There was a more than three-fold increase in monthly household income across the years (*p* for trend < 0.001). There was also an increasing trend in the proportion of non-manual jobs (*p* for trend < 0.001) and the population living in urban areas (*p* for trend < 0.001).

### 3.2. Trends in Alcohol Consumption

**Figure 1:**
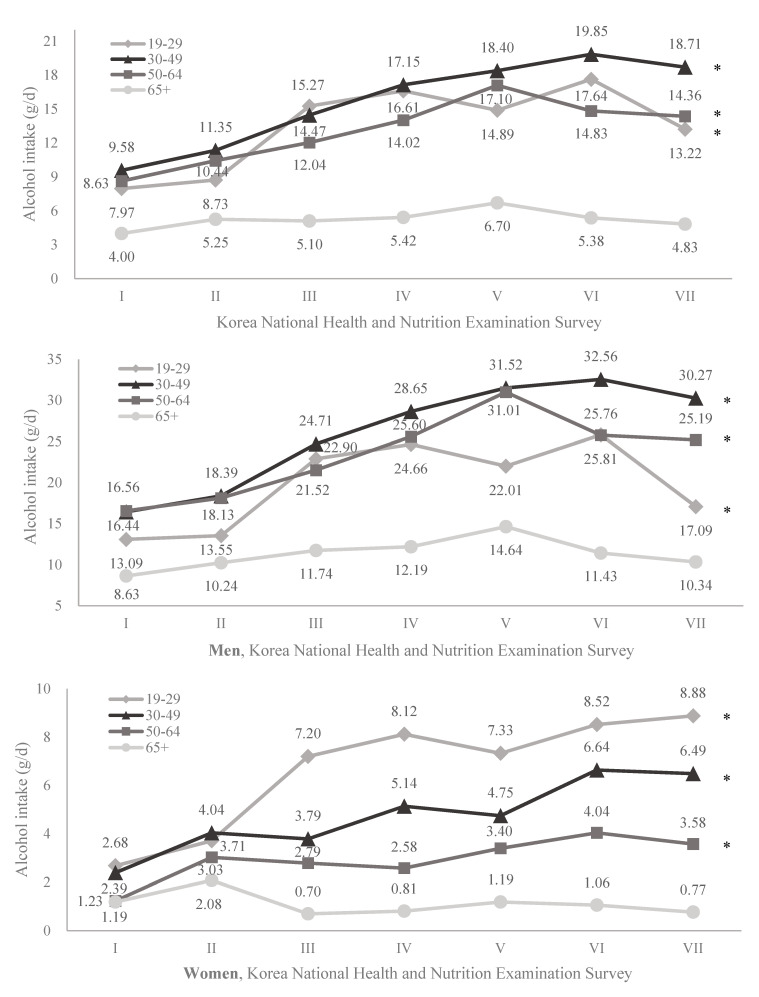
Changes in age-standardized pure alcohol intake by age group over time for Korean adults using the Korea National Health and Nutrition Examination Survey (KNHANES) (* *p* for trend < 0.05).

Trends in daily mean alcohol intake are detailed in Table 2. Pure alcohol consumption significantly increased from 8.37 g/d in 1998 to 14.98 g/d in 2016–2018 (*p* for trend < 0.001). There was an increase of less than double in alcohol intake for men over time from 14.78 g/d to 23.94 g/d (*p* for trend < 0.001). For women, alcohol consumption was significantly lower than men, but escalated more than two times from 2.09 g/d to 5.79 g/d (*p* for trend < 0.001). Figure 1 shows alcohol consumption trends according to age category. An increasing trend was observed for all age groups (*p* for trends < 0.001, respectively) except for the oldest group, ≥65 years (*p* for trend = 0.428). The 30–49 age group tended to drink the most alcohol, and those aged ≥ 65 years consumed the least. However, for women, the greatest amount of alcohol consumption was observed in the 19–29 age group. Additionally, the highest degree of the increasing trend was found for this group as compared to the other age groups.

As shown in Table 2, there was a significant increasing trend in daily alcohol intake regardless of education level, income status, occupation, and region from 1998 to 2018, except for unemployed men. In more detail, alcohol consumption increased the most in both men and women with ≥college education, non-manual occupations, or living in urban areas. However, the amount of alcohol intake increased the most among men with high income and women with middle-high income.

### 3.3. Trends in Alcohol Consumption by Beverage Types

Table 3 presents the trends in alcohol consumption according to seven different types of alcoholic beverage from 1998 to 2018. Daily alcohol intake from beer, makgeolli, wine, soju, and liquor significantly increased over time. In more detail, alcohol consumption from beer escalated (1.38 g/d in 1998 to 3.66 g/d in 2016–2018, *p* for trend < 0.001), makgeolli (0.49 g/d to 0.85 g/d, *p* for trend < 0.001), wine (0.06 g/d to 0.16 g/d, *p* for trend < 0.001), soju (6.08 g/d to 9.95 g/d, *p* for trend < 0.001), and liquor (0.22 g/d to 0.26 g/d, *p* for trend = 0.024). Yet, the trend in alcohol intake from cheongju did not change over time (*p* for trend = 0.226). In men, soju consumption, contributing the highest percentage to the total alcohol consumption, was a peak in 2007–2009, and then a decreasing trend was found from 2007–2009 to 2016–2018 (*p* for trend = 0.035, data not shown).

Among the beverage types, pure alcohol intake from soju was the highest followed by beer across all the years. Additionally, makgeolli was the third-highest consumption, followed by liquor, but its contribution to the total alcohol intake was considerably lower than that of soju and beer. Although pure alcohol intake from soju increased from 1998 to 2018, the percentage of alcohol consumption among the total alcohol intake decreased from 72.73% to 66.47%. The proportion of beer increased from 16.51% to 24.45%, but makgeolli and liquor decreased.

## 4. Discussion

The trend in total alcohol consumption was found to increase (14.98 g in 2016–2018/8.37 g in 1998 = 1.8 times) over time from 1998 to 2018. The amount of alcohol intake among men was incomparably higher than that of women; however, the degree of increase was higher for women (5.79 g in 2016–2018/2.09 g in 1998 = 2.8 times) than for men (23.94 g in 2016–2018/14.78 g in 1998 = 1.6 times). In addition, men aged 30–49 years and women aged 19–29 years consumed the most alcohol and revealed the highest increasing trend (30.27 g in 2016–2018/16.44 g in 1998 = 1.8 times and 8.88 g in 2016–2018/2.68 g in 1998 = 3.3 times, respectively) as compared to the other age groups. Moreover, trends in alcohol consumption according to socioeconomic factors were different. Lastly, alcohol consumption from soju and beer was the highest in terms of total alcohol intake and showed increasing trends from 1998 to 2018, respectively.

Daily pure alcohol intake in both men and women increased from 1998 to 2018. Men consumed a noticeably higher amount of alcohol than women, which may be due to biological differences that women are more vulnerable to the impact of ethanol than men [10]. Additionally, other reasons may be that women have more responsibility for childcare, and men spend more time outside the home, such as working late or socializing in the evening. Lee et al. (2019) provided guidelines for moderate alcohol consumption for Korean adults ≤65 years of ≤8 drinks (112 g) for men and ≤4 drinks (56 g) for women per week [11]. This can be converted to daily alcohol consumption of 16 g for men and 8 g for women. The present study revealed that drinkers who exceeded the criteria were 82.62% in men and 87.38% in women in 2018 (data not shown). Therefore, men may experience alcohol-related problems more frequently than women.

However, the degree of the increasing trend in alcohol consumption among women was greater than that of men and consistently increased from 1998 to 2018, which could be affected by several factors. First, as shown in the results of the present study, education status among women significantly increased, which can explain why opportunities for women to work have increased. Women consumed significantly more alcohol over time, which may be due to social gatherings. In addition, there was a noticeable increase in the average amount of monthly income, and this economic change may affect women to purchase alcoholic beverages.

There was an increasing trend of alcohol intake from 1998 to 2007–2009 among men (*p* for trend < 0.001); however, the period from 2007–2009 to 2016–2018 did not show any changes (*p* for trend = 0.211, data not shown). The risk of various diseases from alcohol consumption could be higher in men as compared to women [12,13,14]. This may be a reasonable motive for men to pay more attention to alcohol intake and take action to control drinking. Another reason could be the modification of the Labor Standard Law in 2018, which reduced work to a maximum of 52 h per week in Korea. It is plausible that this revision changed the drinking culture, including a significant decrease in the frequency of having a drink with co-workers. Thus, it may have affected the decline in daily alcohol consumption from 27.21 g in 2013–2015 to 23.94 g in 2016–2018 (26.12 g in 2016, 24.45 g in 2017, and 21.27 g in 2018, data not shown).

In addition, it is necessary to focus on alcohol consumption in different age categories. First, middle-aged men aged 30–49 years consumed more alcohol than the other age groups. The average age at which men get their first job has gradually increased due to military service and unemployment problems. This means that men have been involved in active social life from their 30s and have many opportunities to drink alcohol. Similarly, it is notable that women in the 19–29 age group consumed the most alcohol. Women started to drink alcohol at an earlier age, and dramatic socioeconomic transitions in Korea led women to consume higher quantities of alcohol as compared to the past. The decline in alcohol consumption after their 20s is due to becoming a wife and mother [15]. This may be a plausible reason why women aged 30–64 years were found to consume significantly less than those aged 19–29 years. Furthermore, mortality risks from alcohol intake can vary according to age [16]. Thus, the results suggest that the target of interventions for alcohol consumption should be not only the overall population but also specific age categories.

Trends in alcohol consumption can be influenced by socioeconomic characteristics. Individuals with a high level of socioeconomic variables showed the largest increase in alcohol intake trends. This is concordant with the results that the annual drinking rate for high-income adults rose from 78.9% in 2005 to 83.1% in 2018, and the increasing degree was higher than that for low-income people (from 75.0% to 76.5%) [7]. Moreover, a study that included 33 countries found that most countries with higher socioeconomic status tended to have more drinkers [17]. It could be that socially active people receive more opportunities to attend occasions to drink alcohol and have budgets to purchase alcoholic beverages. Thus, it is necessary to intervene in alcohol intake based on the socioeconomic factors of Korean adults.

The increasing trend of overall alcohol intake is an issue. In addition, certain alcoholic drinks could be a more serious problem. A meta-analysis showed that breast cancer risk significantly increased with a high intake of wine, rather than beer and spirits [18]. Soju and beer have the highest levels of consumption among alcoholic beverages in Korea. Fortunately, a positive sign was observed that alcohol from soju decreased from 19.30 g in 2007–2009 to 16.92 g in 2016–2018 among men.

The present study revealed daily alcohol intake from makgeolli showed a stiff increase by more than two times from 2007–2009 to 2010–2012, which was a unique aspect as compared to the results for the other alcoholic beverages. At the end of 2008, there was a makgeolli boom in Korea. One of the reasons was because makgeolli began to be treated as a well-being product that is beneficial for health [19]. We observed a dramatic increase in alcohol consumption from makgeolli, specifically, 0.46 g in 2008, 0.77 g in 2009, and 1.97 g in 2010 (data not shown). After undergoing the boom, it is plausible to suggest that Korean people tend to be concerned about health and pay attention to what they consume.

There has been a constant increase in alcohol consumption over time. This increasing trend can cause numerous public health consequences. In Korea, the prevalence of obesity increased from 36.6% in 2008 to 44.7% in 2018 for adult men [7]. Additionally, the prevalence of non-alcoholic fatty liver disease and alcohol-related liver disease escalated from 1998–2001 to 2016–2017, specifically, from 18.6% to 21.5% and 3.8% to 7.0%, respectively [20]. However, the information related to alcohol guidelines is insufficient and currently unclear. The effect of the Korean government’s anti-smoking policies successfully decreased the prevalence of smoking in men [21]. Similarly, this movement is demanding to apply to decrease alcohol intake as well.

This study has several strengths. It utilized KNHANES data, which represents the Korean population, and used all available data sets from 1998 to 2018. It is critical to examine the actual intake using the 24-h dietary recall, since calculating the daily mean intake of the population has not been suggested via food frequency questionnaires due to large measurement errors [22]. To the best of our knowledge, this study is the first to assess trends in pure alcohol intake through a 24-h dietary recall to show the actual amount of alcohol consumption. A limitation could be the change in the survey protocol from 2007.

## 5. Conclusions

Daily alcohol intake was found to have increased over time from 1998 to 2018 in Korea. Alcohol consumption was higher in men than in women, but the amount of the increasing trend was greater for women than men. Additionally, the highest alcohol intake was reported in men aged 30–49 years and women aged 19–29 years. Moreover, the degree of the alcohol consumption trend tended to increase the most among people with high socioeconomic factors. In addition, trends in alcohol intake by the alcoholic beverage types increased over time, especially in beer and soju, which were the main percentages of the total alcohol intake. Thus, it is critical to provide appropriate education and guidelines to decrease alcohol consumption, considering not only the overall Korean population but also specific characteristics of individuals. Furthermore, concrete policies and regulations of alcohol use should be prepared by different characteristics of the population. Additionally, future investigations are needed to discover associations between the selected alcohol drinks and health outcomes.

## Figures and Tables

**Table 1:** Trends in general characteristics of Korean adults ≥ 19 years from 1998 to 2018 using the Korea National Health and Nutrition Examination Survey (KNHANES).

	KNHANES							
	I (1998)	II (2001)	III (2005)	IV (2007–2009)	V (2010–2012)	VI (2013–2015)	VII (2016–2018)	*p* for Trend ^†^
Total (n)	7501	7092	6526	16,187	17,394	16,069	16,854	
Age (y, mean ± SE)	41.28 ± 0.33	41.82 ± 0.29	43.30 ± 0.30	44.57 ± 0.24	45.53 ± 0.24	46.34 ± 0.23	47.28 ± 0.24	**<0.001**
Age (y, % (SE))								
19–29	26.71 (0.82)	25.54 (0.76)	22.17 (0.82)	20.33 (0.60)	19.08 (0.56)	18.32 (0.52)	17.60 (0.53)	**<0.001**
30–49	45.62 (1.12)	45.89 (0.98)	46.23 (1.02)	44.47 (0.71)	42.50 (0.70)	40.09 (0.69)	38.27 (0.71)	**<0.001**
50–64	18.03 (0.70)	18.18 (0.58)	19.56 (0.62)	21.73 (0.48)	23.96 (0.48)	25.87 (0.49)	27.40 (0.49)	**<0.001**
≥65	9.64 (0.54)	10.39 (0.53)	12.04 (0.57)	13.47 (0.41)	14.46 (0.42)	15.72 (0.43)	16.73 (0.47)	**<0.001**
Education ^‡^ (% (SE))								
≤Middle school	37.42 (0.93)	33.38 (0.80)	28.56 (0.69)	28.00 (0.47)	24.60 (0.40)	20.61 (0.39)	17.49 (0.38)	**<0.001**
High school	37.79 (0.80)	36.88 (0.82)	35.49 (0.87)	41.93 (0.60)	40.68 (0.61)	39.59 (0.63)	36.13 (0.64)	**0.002**
≥College	24.78 (1.12)	29.73 (0.90)	35.95 (1.04)	30.07 (0.72)	34.72 (0.67)	39.80 (0.71)	46.38 (0.74)	**<0.001**
Income ^§^ (10^4^ KRW, mean ± SE)	140.34 ± 3.40	230.94 ± 6.09	234.46 ± 4.89	309.95 ± 9.02	449.30 ± 14.59	394.21 ± 6.14	464.85 ± 6.62	**<0.001**
Occupation ^‡^ (mean (SE))								
Manual	27.53 (0.99)	24.30 (0.80)	26.49 (0.95)	24.64 (0.64)	25.38 (0.67)	21.79 (0.55)	20.66 (0.54)	**<0.001**
Non-manual	32.01 (0.91)	34.73 (0.91)	34.51 (0.81)	35.77 (0.61)	38.82 (0.62)	41.73 (0.59)	44.59 (0.59)	**<0.001**
Unemployed	40.46 (0.72)	40.97 (0.76)	39.00 (0.74)	39.59 (0.59)	35.80 (0.58)	36.48 (0.52)	34.75 (0.54)	**<0.001**
Region ^‡^ (mean (SE))								
Urban	76.54 (1.18)	79.80 (0.88)	81.54 (1.11)	81.35 (1.52)	80.75 (1.60)	82.91 (1.48)	85.82 (1.40)	**<0.001**
Rural	23.46 (1.18)	20.20 (0.88)	18.46 (1.11)	18.65 (1.52)	19.25 (1.60)	17.09 (1.48)	14.18 (1.40)	**<0.001**
Men (n)	3480	3253	2918	6592	7129	6712	7165	
Age (y, mean ± SE)	40.25 ± 0.32	40.86 ± 0.32	42.30 ± 0.36	43.55 ± 0.27	44.51 ± 0.29	45.25 ± 0.26	46.17 ± 0.28	**<0.001**
Age (y, % (SE))								
19–29	27.57 (1.00)	26.41 (1.00)	23.02 (1.15)	21.13 (0.83)	19.91 (0.78)	19.53 (0.73)	18.74 (0.73)	**<0.001**
30–49	47.38 (1.34)	47.34 (1.18)	47.73 (1.30)	45.97 (0.91)	43.96 (0.86)	41.28 (0.88)	39.52 (0.88)	**<0.001**
50–64	17.66 (0.76)	18.07 (0.71)	19.54 (0.78)	21.85 (0.60)	24.12 (0.61)	25.97 (0.63)	27.47 (0.66)	**<0.001**
≥65	7.39 (0.54)	8.18 (0.52)	9.72 (0.62)	11.05 (0.42)	12.01 (0.43)	13.22 (0.43)	14.27 (0.48)	**<0.001**
Education ^‡^ (% (SE))								
≤Middle school	29.37 (1.02)	26.73 (0.93)	22.26 (0.80)	23.32 (0.61)	20.02 (0.49)	16.98 (0.50)	14.30 (0.47)	**<0.001**
High school	40.77 (1.09)	37.13 (1.03)	35.80 (1.11)	43.24 (0.83)	42.81 (0.78)	41.46 (0.89)	37.13 (0.80)	**<0.001**
≥College	29.86 (1.37)	36.13 (1.10)	41.93 (1.27)	33.44 (0.91)	37.17 (0.82)	41.56 (0.94)	48.57 (0.90)	**<0.001**
Income ^§^ (10^4^ KRW, mean ± SE)	139.85 ± 3.42	231.27 ± 6.46	233.91 ± 4.94	307.03 ± 8.17	442.30 ± 16.14	398.09 ± 6.75	468.23 ± 7.26	**<0.001**
Occupation ^‡^ (mean (SE))								
Manual	37.19 (1.25)	33.78 (1.09)	36.98 (1.20)	34.91 (0.94)	35.48 (0.93)	31.47 (0.85)	30.07 (0.83)	**<0.001**
Non-manual	37.68 (1.29)	39.64 (1.14)	37.18 (1.10)	40.51 (0.91)	41.59 (0.95)	44.34 (0.89)	45.94 (0.85)	**<0.001**
Unemployed	25.12 (0.93)	26.58 (0.87)	25.84 (0.82)	24.58 (0.71)	22.93 (0.70)	24.19 (0.66)	23.99 (0.62)	0.057
Region ^‡^ (mean (SE))								
Urban	76.03 (1.23)	79.29 (1.05)	80.81 (1.33)	80.62 (1.60)	79.88 (1.72)	81.67 (1.60)	85.24 (1.46)	**<0.001**
Rural	23.97 (1.23)	20.71 (1.05)	19.19 (1.33)	19.38 (1.60)	20.12 (1.72)	18.33 (1.60)	14.76 (1.46)	**<0.001**
Women (n)	4021	3839	3608	9595	10,265	9357	9689	
Age (y, mean ± SE)	42.29 ± 0.41	42.76 ± 0.38	44.28 ± 0.35	45.57 ± 0.27	46.52 ± 0.27	47.41 ± 0.27	48.36 ± 0.28	**<0.001**
Age (y, % (SE))								
19–29	25.88 (0.95)	24.70 (0.92)	21.33 (0.93)	19.55 (0.67)	18.26 (0.62)	17.14 (0.59)	16.48 (0.57)	**<0.001**
30–49	43.91 (1.16)	44.47 (1.03)	44.77 (1.08)	43.00 (0.76)	41.08 (0.73)	38.91 (0.71)	37.03 (0.74)	**<0.001**
50–64	18.40 (0.82)	18.29 (0.68)	19.58 (0.74)	21.61 (0.52)	23.81 (0.54)	25.77 (0.54)	27.33 (0.54)	**<0.001**
≥65	11.81 (0.68)	12.55 (0.68)	14.32 (0.69)	15.83 (0.51)	16.85 (0.50)	18.18 (0.54)	19.16 (0.58)	**<0.001**
Education ^‡^ (% (SE))								
≤Middle school	44.80 (1.02)	39.43 (0.88)	34.16 (0.80)	32.04 (0.51)	28.51 (0.49)	23.60 (0.43)	20.15 (0.42)	**<0.001**
High school	35.42 (0.93)	37.10 (0.93)	35.78 (1.04)	40.83 (0.67)	38.71 (0.77)	37.67 (0.74)	35.15 (0.78)	**0.002**
≥College	19.78 (1.05)	23.46 (0.91)	30.06 (1.14)	27.14 (0.74)	32.78 (0.78)	38.73 (0.81)	44.70 (0.84)	**<0.001**
Income ^‡,§^ (10^4^ KRW, mean ± SE)	140.75 ± 3.52	231.03 ± 6.38	235.39 ± 5.43	313.27 ± 11.40	457.83 ± 15.12	391.06 ± 6.45	462.65 ± 7.02	**<0.001**
Occupation ^‡^ (mean (SE))								
Manual	18.27 (0.97)	14.99 (0.80)	16.15 (0.91)	14.77 (0.54)	15.91 (0.63)	12.77 (0.51)	11.44 (0.47)	**<0.001**
Non-manual	26.45 (0.96)	29.88 (1.09)	31.98 (0.95)	31.37 (0.67)	36.35 (0.70)	39.68 (0.72)	43.49 (0.75)	**<0.001**
Unemployed	55.29 (1.11)	55.13 (1.10)	51.88 (1.10)	53.86 (0.76)	47.74 (0.73)	47.55 (0.72)	45.07 (0.78)	**<0.001**
Region ^‡^ (mean (SE))								
Urban	76.97 (1.19)	80.35 (0.82)	82.29 (1.02)	82.13 (1.49)	81.68 (1.56)	84.22 (1.40)	86.47 (1.41)	**<0.001**
Rural	23.03 (1.19)	19.65 (0.82)	17.71 (1.02)	17.87 (1.49)	18.32 (1.56)	15.78 (1.40)	13.53 (1.41)	**<0.001**

**Bold** values indicate *p* for trends < 0.05. ^†^ *p* for trends by multivariate linear regression for continuous variables (age and income) or logistic regression for categorical variables (each category of age, education, occupation, and region). ^‡^ Age-standardized based on the 2005 Korea census, and *p* for trends adjusting for age. ^§^ Household monthly income in Korean won (approximately 1082 Korean won equal to 1 U.S. dollar in 2018).

**Table 2:** Trends in daily consumption of pure alcohol for Korean adults aged ≥ 19 years from 1998 to 2018 using the Korea National Health and Nutrition Examination Survey (KNHANES).

	KNHANES							
	I (1998)	II (2001)	III (2005)	IV (2007–2009)	V (2010–2012)	VI (2013–2015)	VII (2016–2018)	*p* for Trend ^†^
Total (n)	7501	7092	6526	16,187	17,394	16,069	16,854	
Alcohol intake ^‡^ (g/d, mean ± SE)	8.37 ± 0.49	9.86 ± 0.57	13.07 ± 0.83	15.03 ± 0.52	15.97 ± 0.59	16.67 ± 0.53	14.98 ± 0.49	**<0.001**
Alcohol intake by education ^‡^ (g/d, mean ± SE)								
≤Middle school	11.55 ± 1.76	11.68 ± 1.81	14.08 ± 2.89	19.19 ± 3.82	21.05 ± 5.37	20.64 ± 4.12	15.68 ± 1.73	**<0.001**
High school	9.35 ± 0.90	10.88 ± 1.11	13.70 ± 1.25	15.68 ± 0.76	16.88 ± 0.92	17.35 ± 0.91	15.08 ± 0.77	**<0.001**
≥College	10.14 ± 1.27	10.51 ± 1.05	14.87 ± 1.46	15.96 ± 1.43	16.03 ± 0.89	15.67 ± 0.91	14.81 ± 0.70	**<0.001**
Alcohol intake by income ^‡^ (g/d, mean ± SE)								
Low	9.54 ± 0.90	10.00 ± 0.95	14.10 ± 1.55	14.90 ± 1.00	15.37 ± 1.23	15.02 ± 1.01	13.23 ± 0.94	**<0.001**
Middle-low	7.83 ± 0.96	9.92 ± 0.97	12.36 ± 1.66	16.10 ± 1.07	16.42 ± 1.21	16.42 ± 1.06	14.69 ± 0.92	**<0.001**
Middle-high	9.83 ± 1.04	10.99 ± 1.24	12.92 ± 1.28	14.46 ± 0.93	16.49 ± 1.08	17.01 ± 1.14	16.22 ± 1.04	**<0.001**
High	6.54 ± 0.78	9.00 ± 0.84	12.48 ± 1.50	15.14 ± 1.06	15.76 ± 0.94	18.32 ± 1.18	16.02 ± 1.04	**<0.001**
Alcohol intake by occupation ^‡^ (g/d, mean ± SE)								
Manual	11.49 ± 0.94	15.74 ± 1.49	18.09 ± 1.77	20.48 ± 1.49	20.73 ± 1.45	24.68 ± 1.82	17.98 ± 1.28	**<0.001**
Non-manual	9.44 ± 0.96	10.49 ± 1.00	15.62 ± 1.82	18.37 ± 1.16	18.21 ± 0.97	16.93 ± 0.87	16.67 ± 0.77	**<0.001**
Unemployed	4.89 ± 0.57	5.39 ± 0.54	7.33 ± 0.79	7.76 ± 0.54	7.09 ± 0.67	8.74 ± 0.67	7.95 ± 0.58	**<0.001**
Alcohol intake by region ^‡^ (g/d, mean ± SE)								
Urban	7.87 ± 0.56	9.74 ± 0.64	12.66 ± 0.88	14.64 ± 0.58	15.66 ± 0.63	16.31 ± 0.57	14.94 ± 0.53	**<0.001**
Rural	10.17 ± 1.18	9.89 ± 1.04	14.71 ± 1.99	16.65 ± 1.35	16.97 ± 1.58	18.08 ± 1.35	14.67 ± 1.27	**<0.001**
Men (n)	3480	3253	2918	6592	7129	6712	7165	
Alcohol intake ^‡^ (g/d, mean ± SE)	14.78 ± 0.92	16.28 ± 0.98	22.15 ± 1.49	25.21 ± 0.98	27.27 ± 1.08	27.21 ± 0.94	23.94 ± 0.89	**<0.001**
Alcohol intake by education ^‡^ (g/d, mean ± SE)								
≤Middle school	21.39 ± 2.65	20.08 ± 2.97	26.41 ± 4.79	38.34 ± 8.16	42.88 ±12.63	31.88 ± 5.54	25.89 ± 3.35	**<0.001**
High school	14.73 ± 1.35	17.51 ± 1.84	21.58 ± 2.07	25.15 ± 1.37	28.42 ± 1.68	28.87 ± 1.73	25.22 ± 1.44	**<0.001**
≥College	13.72 ± 1.74	14.34 ± 1.41	21.39 ± 2.07	24.84 ± 2.24	25.20 ± 1.59	25.16 ± 1.70	22.41 ± 1.26	**<0.001**
Alcohol intake by income ^‡^ (g/d, mean ± SE)								
Low	16.92 ± 1.56	16.25 ± 1.61	22.60 ± 2.79	24.71 ± 1.80	26.73 ± 2.35	24.73 ± 1.78	20.38 ± 1.68	**0.002**
Middle-low	13.88 ± 1.83	15.94 ± 1.69	20.87 ± 3.00	26.55 ± 1.94	27.63 ± 2.23	26.35 ± 1.86	23.76 ± 1.64	**<0.001**
Middle-high	17.15 ± 1.90	18.77 ± 2.29	22.03 ± 2.41	24.96 ± 1.77	28.17 ± 2.02	27.33 ± 2.05	25.84 ± 1.86	**<0.001**
High	11.54 ± 1.53	14.72 ± 1.48	22.48 ± 2.63	25.39 ± 1.98	26.63 ± 1.73	30.73 ± 2.06	26.14 ± 1.85	**<0.001**
Alcohol intake by occupation ^‡^ (g/d, mean ± SE)								
Manual	15.69 ± 1.23	19.11 ± 1.73	23.87 ± 2.19	26.82 ± 1.80	27.91 ± 2.01	31.32 ± 2.22	22.61 ± 1.63	**<0.001**
Non-manual	14.16 ± 1.56	15.29 ± 1.49	25.24 ± 3.57	28.22 ± 2.07	30.15 ± 1.88	26.83 ± 1.67	25.85 ± 1.41	**<0.001**
Unemployed	13.71 ± 1.87	13.01 ± 1.97	18.82 ± 3.37	21.05 ± 2.48	17.25 ± 2.11	16.98 ± 2.70	17.33 ± 2.26	0.059
Alcohol intake by region ^‡^ (g/d, mean ± SE)								
Urban	13.71 ± 1.06	16.18 ± 1.14	21.53 ± 1.61	24.53 ± 1.10	26.75 ± 1.15	27.06 ± 1.03	23.89 ± 0.96	**<0.001**
Rural	18.40 ± 2.07	16.18 ± 1.69	23.92 ± 3.35	28.00 ± 2.34	28.78 ± 2.80	26.92 ± 2.19	23.35 ± 2.23	**<0.001**
Women (n)	4021	3839	3608	9595	10,265	9357	9689	
Alcohol intake ^‡^ (g/d, mean ± SE)	2.09 ± 0.23	3.54 ± 0.39	4.00 ± 0.41	4.81 ± 0.27	4.65 ± 0.26	5.90 ± 0.33	5.79 ± 0.30	**<0.001**
Alcohol intake by education ^‡^ (g/d, mean ± SE)								
≤Middle school	2.95 ± 0.68	3.94 ± 0.74	6.39 ± 3.34	5.63 ± 1.40	6.09 ± 1.40	12.13 ± 6.28	7.07 ± 1.47	**0.001**
High school	1.85 ± 0.34	3.00 ± 0.55	5.11 ± 0.77	5.83 ± 0.47	4.56 ± 0.39	5.95 ± 0.45	5.81 ± 0.48	**<0.001**
≥College	1.53 ± 0.34	3.02 ± 1.03	2.68 ± 0.60	2.89 ± 0.31	3.94 ± 0.37	4.61 ± 0.40	5.45 ± 0.42	**<0.001**
Alcohol intake by income ^‡^ (g/d, mean ± SE)								
Low	2.20 ± 0.61	4.18 ± 0.89	5.26 ± 1.03	4.64 ± 0.55	4.47 ± 0.51	5.00 ± 0.55	6.02 ± 0.54	**0.004**
Middle-low	2.06 ± 0.37	3.77 ± 0.94	4.08 ± 0.71	5.45 ± 0.60	4.58 ± 0.52	6.10 ± 0.67	5.63 ± 0.56	**<0.001**
Middle-high	2.23 ± 0.45	3.17 ± 0.53	3.96 ± 0.74	4.16 ± 0.43	4.81 ± 0.59	6.57 ± 0.82	6.03 ± 0.65	**<0.001**
High	1.94 ± 0.35	3.19 ± 0.59	2.66 ± 0.53	4.70 ± 0.51	4.84 ± 0.55	5.89 ± 0.59	5.42 ± 0.58	**<0.001**
Alcohol intake by occupation ^‡^ (g/d, mean ± SE)								
Manual	2.22 ± 0.54	2.50 ± 0.44	2.58 ± 0.52	4.47 ± 1.13	5.62 ± 0.93	8.15 ± 3.23	5.30 ± 1.15	**0.038**
Non-manual	2.14 ± 0.45	3.50 ± 0.54	4.43 ± 0.59	5.96 ± 0.45	5.31 ± 0.41	6.07 ± 0.40	6.61 ± 0.48	**<0.001**
Unemployed	1.80 ± 0.25	2.71 ± 0.41	3.75 ± 0.63	4.11 ± 0.40	3.42 ± 0.38	4.89 ± 0.43	4.85 ± 0.50	**<0.001**
Alcohol intake by region ^‡^ (g/d, mean ± SE)								
Urban	2.15 ± 0.27	3.44 ± 0.40	3.94 ± 0.42	4.89 ± 0.30	4.78 ± 0.28	5.70 ± 0.36	5.88 ± 0.33	**<0.001**
Rural	1.73 ± 0.34	3.17 ± 0.72	4.66 ± 1.49	4.07 ± 0.58	3.73 ± 0.58	7.22 ± 0.93	4.91 ± 0.80	**<0.001**

**Bold** values indicate *p* for trends < 0.05. ^†^ *p* for trends by multivariate linear regression. ^‡^ Age-standardized based on the 2005 Korean census, and *p* for trends adjusting for age.

**Table 3:** Trends in daily consumption of pure alcohol by different types of alcohol for Korean adults aged ≥ 19 years from 1998 to 2018 using the Korea National Health and Nutrition Examination Survey (KNHANES).

	KNHANES							
	Ⅰ (1998)	Ⅱ (2001)	Ⅲ (2005)	Ⅳ (2007–2009)	Ⅴ (2010–2012)	Ⅵ (2013–2015)	Ⅶ (2016–2018)	*p* for Trend ^†^
Total (n)	7501	7092	6526	16,187	17,394	16,069	16,854	
Alcohol intake ^‡^ (g/d, mean ± SE)								
Total	8.37 ± 0.49	9.86 ± 0.57	13.07 ± 0.83	15.03 ± 0.52	15.97 ± 0.59	16.67 ± 0.53	14.98 ± 0.49	**<0.001**
Beer	1.38 ± 0.13	1.69 ± 0.18	2.42 ± 0.24	2.87 ± 0.15	3.59 ± 0.18	3.79 ± 0.17	3.66 ± 0.15	**<0.001**
Makgeolli ^§^	0.49 ± 0.08	0.42 ± 0.11	0.74 ± 0.14	0.61 ± 0.06	1.56 ± 0.11	1.40 ± 0.12	0.85 ± 0.07	**<0.001**
Wine	0.06 ± 0.02	0.05 ± 0.02	0.07 ± 0.03	0.16 ± 0.03	0.12 ± 0.02	0.16 ± 0.04	0.16 ± 0.03	**<0.001**
Cheongju ^§^	0.08 ± 0.04	0.04 ± 0.02	0.15 ± 0.05	0.12 ± 0.03	0.18 ± 0.05	0.12 ± 0.03	0.08 ± 0.02	0.226
Soju ^§^	6.08 ± 0.38	7.46 ± 0.47	9.32 ± 0.63	10.86 ± 0.45	9.97± 0.45	10.83 ± 0.44	9.95 ± 0.40	**<0.001**
Liquor	0.22 ± 0.07	0.11 ± 0.04	0.32 ± 0.09	0.40 ± 0.08	0.52 ± 0.15	0.33 ± 0.07	0.26 ± 0.06	**0.024**
Other ^§^	0.05 ± 0.04	0.09 ± 0.04	0.06 ± 0.04	0.01 ± 0.01	0.03 ± 0.01	0.04 ± 0.01	0.01 ± 0.01	0.101
Men (n)	3480	3253	2918	6592	7129	6712	7165
Alcohol intake ^‡^ (g/d, mean ± SE)								
Total	14.78 ± 0.92	16.28± 0.98	22.15 ± 1.49	25.21 ± 0.98	27.27 ± 1.08	27.21 ± 0.94	23.94 ± 0.89	**<0.001**
Beer	2.07 ± 0.23	2.39 ± 0.30	3.25 ± 0.41	3.90 ± 0.24	5.33 ± 0.30	5.41 ± 0.29	4.84 ± 0.26	**<0.001**
Makgeolli ^§^	0.92 ± 0.17	0.61 ± 0.14	1.39 ± 0.28	0.98 ± 0.11	2.66 ± 0.20	2.42 ± 0.23	1.42 ± 0.12	**<0.001**
Wine	0.09 ± 0.04	0.07 ± 0.03	0.06 ± 0.04	0.18 ± 0.06	0.13 ± 0.03	0.22 ± 0.06	0.19 ± 0.04	**0.002**
Cheongju ^§^	0.16 ± 0.07	0.05 ± 0.03	0.26 ± 0.09	0.16 ± 0.05	0.28 ± 0.09	0.18 ± 0.06	0.11 ± 0.04	0.569
Soju ^§^	11.17 ± 0.72	12.85 ± 0.83	16.53 ± 1.16	19.30 ± 0.86	17.86 ± 0.84	18.37 ± 0.79	16.92 ± 0.73	**<0.001**
Liquor	0.28 ± 0.11	0.15 ± 0.05	0.56 ± 0.18	0.69 ± 0.16	0.99 ± 0.29	0.57 ± 0.13	0.46 ± 0.10	**0.004**
Other ^§^	0.09 ± 0.08	0.15 ± 0.07	0.10 ± 0.08	0.01 ± 0.01	0.03 ± 0.02	0.04 ± 0.02	0.01 ± 0.01	0.072
Women (n)	4021	3839	3608	9595	10,265	9357	9689
Alcohol intake ^‡^ (g/d, mean ± SE)								
Total	2.09 ± 0.23	3.54 ± 0.39	4.00 ± 0.41	4.81 ± 0.27	4.65 ± 0.26	5.90 ± 0.33	5.79 ± 0.30	**<0.001**
Beer	0.68 ± 0.09	0.96 ± 0.13	1.57 ± 0.24	1.84 ± 0.14	1.80 ± 0.13	2.09 ± 0.12	2.45 ± 0.14	**<0.001**
Makgeolli ^§^	0.07 ± 0.02	0.23 ± 0.10	0.13 ± 0.03	0.25 ± 0.04	0.50 ± 0.06	0.39 ± 0.05	0.30 ± 0.04	**<0.001**
Wine	0.03 ± 0.01	0.02 ± 0.01	0.08 ± 0.03	0.14 ± 0.04	0.12 ± 0.04	0.10 ± 0.02	0.13 ± 0.03	**<0.001**
Cheongju ^§^	0.01 ± 0.01	0.03 ± 0.02	0.04 ± 0.01	0.09 ± 0.04	0.08 ± 0.03	0.05 ± 0.02	0.06 ± 0.03	**0.047**
Soju ^§^	1.11 ± 0.16	2.20 ± 0.30	2.10 ± 0.29	2.38 ± 0.19	2.09 ± 0.21	3.16 ± 0.27	2.78 ± 0.23	**<0.001**
Liquor	0.17 ± 0.08	0.08 ± 0.05	0.06 ± 0.03	0.09 ± 0.03	0.04 ± 0.02	0.08 ± 0.03	0.05 ± 0.02	0.206
Other ^§^	0.02 ± 0.01	0.02 ± 0.01	0.02 ± 0.01	0.02 ± 0.01	0.02 ± 0.01	0.03 ± 0.02	0.02 ± 0.01	0.599

**Bold** values indicate *p* for trends < 0.05. ^†^ *p* for trends by multivariate linear regression. ^‡^ Age-standardized based on the 2005 Korean census, and *p* for trends adjusting for age. ^§^ Makgeolli as a traditional Korean rice wine, cheongju as a traditional Korean clear refined rice wine, soju as a Korean distilled spirit, and other including cocktail and champagne.

## Data Availability

The data presented in this study are available at http://knhanes.cdc.go.kr/.
